# Response properties of spiking and non-spiking brain neurons mirror pulse interval selectivity

**DOI:** 10.3389/fncel.2022.1010740

**Published:** 2022-09-29

**Authors:** Xinyang Zhang, Berthold Hedwig

**Affiliations:** Department of Zoology, University of Cambridge, Cambridge, United Kingdom

**Keywords:** auditory processing, pattern recognition, brain neuron, delay-line, template matching

## Abstract

In the bispotted field cricket auditory pulse pattern recognition of the species-specific calling song is based on a delay-line and coincidence detection network, established by the activity and synaptic connections of only 5 auditory neurons in the brain. To obtain a more detailed understanding of the network and the dynamic of the neural activity over time we analyzed the response properties of these neurons to test patterns, in which the pulse duration was kept constant while the duration of specific pulse intervals was systematically altered. We confirm that the ascending interneuron AN1 and the local interneuron LN2 copy the structure of the pulse pattern, however with limited resolution at short pulse intervals, further evident in downstream neural responses. In the non-spiking delay-line interneuron LN5 during long pulse intervals full-blown rebound potentials develop over a time course of 35–70 ms. LN5 also reveals an overall increase in its membrane potential tuned to chirps of the calling song pulse pattern. This may contribute to the pattern recognition process by driving the activity of the coincidence-detector LN3 and may indicate a further function of the delay-line neuron LN5. The activity of LN3 and of the feature detector LN4 match the tuning of the phonotactic behavior and demonstrate an increasingly sparse coding of the calling song pulse patterns as evident in the response of the feature detector LN4. The circuitry reveals a fundamental mechanism of auditory pattern recognition and demonstrates a principle of neuronal coding.

## Highlights

- Analyzing the processing of temporal patterns by brain interneurons and circuitry in cricket has provided a comprehensive understanding of pattern recognition in animals.
- We analyzed the response properties of the phonotactic behavior and the identified neural circuitry to chirps with a constant duration of sound pulses while varying the durations of specific pulse intervals.
- Our results replicate and expand the scope of existing research reported previously.

- We showed that the phonotactic response under our research conditions also tuned specifically to the chirps close to the animal’s natural calling song.
- Using intracellular recordings, we revealed the functional properties of individual neurons in the network in more detail.
- We analyzed the change in neuronal activities in response to the change of a single interval of a sound.
- Several novel findings by our investigation are worth emphasizing.
- Even a change of a single interval within a chirp can significantly alter the behavioral and neural response toward the sound, which was not reported before.
- We further demonstrated that the non-spiking neuron not only serves as a delay-line for coincidence detection but also demonstrates intrinsic response properties already tuned to pattern recognition at this early stage of the processing.
- This expands the previously described function and point toward an adapted neuronal filter mechanism at the level of an individual neuron for the processing of a species-specific auditory pulse pattern.
- These findings provided insights to broader studies on pattern recognition in both invertebrates and vertebrates.

## Introduction

Auditory pathways in vertebrates and invertebrates face similar functional challenges (Albert and Kozlov, 2016). While hearing organs provide the CNS with afferent information about sound intensity and frequency, the decoding of temporal patterns must be achieved in the central nervous system (Hildebrandt, 2014; Kostarakos and Hedwig, 2015). For the processing of temporal pulse patterns coincidence detection mechanisms have been suggested and described in vertebrates (Langner, 1992; Crawford, 1997; Large and Crawford, 2002; Covey and Faure, 2005; Rose, 2014) and invertebrates (Weber and Thorson, 1989; Schöneich et al., 2015). Crucial to the function of these circuits are neurons that upon stimulation with a sound pulse respond with an initial hyperpolarization followed a post-inhibitory rebound (PIR), timed to the temporal features of the acoustic signal. This allows delaying an excitatory response over an interval of many milliseconds to be integrated and processed with subsequent auditory responses at the level of a coincidence detector that also receives a direct input from the auditory pathway. This processing mechanism provides the basis for tuning to pulse sequences with a timing matching the delay of the PIR.

In the bispotted field cricket *Gryllus bimaculatus* the processing of pulse interval and pulse duration is crucial for the recognition of the species-specific song pattern. For the processing of the sound envelope a delay-line and coincidence-detector mechanism composed of a network of few brain neurons has been identified for the recognition of the species-specific pulse pattern (Kostarakos and Hedwig, 2012; Schöneich et al., 2015; Hedwig and Sarmiento-Ponce, 2017). Evidence is based on a non-spiking interneuron (LN5) that upon stimulation with a sound pulse responds with an inhibition followed by a delayed PIR with a time course matching the pulse period of the calling song. In a subsequent coincidence detector (LN3) this delayed graded excitation is integrated with the directly forwarded spike activity of an ascending interneuron (AN1), copying the pulse pattern. The response of the coincidence detector is boosted when both the delayed response and the direct response of the ascending neuron coincide; the output of the coincidence detector and an inhibitory input drive the activity of a feature detector (LN4). Both, the tuning of the coincidence detector neuron and of the feature detector neuron match the tuning of the phonotactic behavior (Kostarakos and Hedwig, 2012; Schöneich et al., 2015). Details of the circuitry’s performance have been tested and supported in behavioral experiments (Hedwig and Sarmiento-Ponce, 2017) and have been analyzed in a comprehensive computational modeling study (Clemens et al., 2021).

The non-spiking neuron so far has only been considered as a delay-line in the recognition circuit. Here we systematically analyzed the time course of the neural responses and of the PIR by varying the interval between two adjacent sound pulses, while keeping the pulse duration constant. In this way we aim to explore the time course and dynamics of the PIR and its functional relevance for pattern recognition (Hedwig and Sarmiento-Ponce, 2017).

Our results furthermore confirm the properties of the pattern recognition network and point toward an adapted neuronal filter mechanism at the level of an individual neuron for the processing of a species-specific auditory pulse pattern. We provide evidence that the non-spiking neuron not only provides a delay-line for coincidence detection but also demonstrates intrinsic response properties that mirror the tuning of phonotactic behavior, this expands the previously described function of LN5 (Schöneich et al., 2015).

## Materials and methods

### Animals

Last instar nymphs of *Gryllus bimaculatus* DeGeer were separated from our colony at the Department of Zoology/Cambridge and kept individually in plastic containers at 28°C with a 12/12h light/dark cycle, isolated from singing males, and provided *ad libitum* with food and water. Adult female crickets at 10–25 days post-ecdysis with intact tympanal membranes and spiracles were selected. Experiments were performed at 23–24°C.

### Acoustic stimulation

Sound stimuli were designed with Cool Edit Pro 2000 software (Syntrillium, Phoenix, AZ, USA) and delivered by a PC. Stimuli were presented by two speakers (Sinus live NEO 13 s, Conrad Electronics, Hirschau, Germany) placed frontal to the cricket at an angle of 45° from the left and the right to the animal’s long axis. Carrier frequency was set to 4.8 kHz, the rising and falling ramps of sound pulses were 2 ms. Sound intensity was calibrated to 75 dB SPL, relative to 20 μPa at the location of the cricket using a Brüel and Kjaer measuring amplifier and a 1/2-inch free field microphone (models 2610 and 4939, respectively; Naerum, Denmark).

**FIGURE 1:**
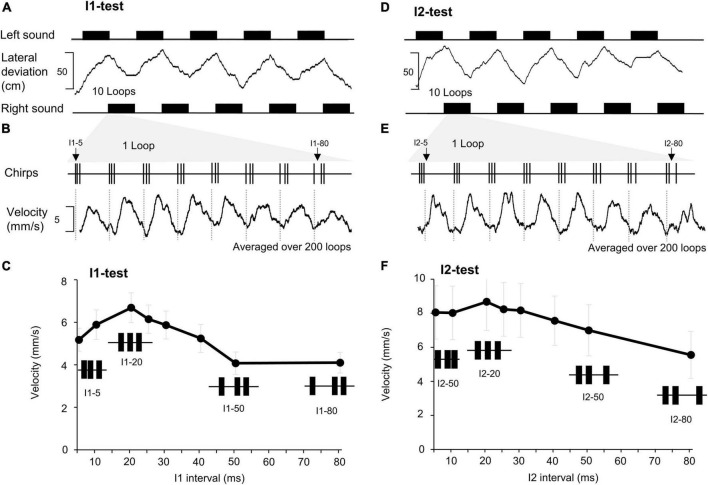
Effect of changing the first and the second intervals of a chirp on phonotaxis. **(A)** A total of 10 sound sequences, each containing 10 loops of the I1-chirps were presented from the left and right side (black rectangles), for each side 5 sequences are shown. The lateral deviation (middle) indicates the phonotactic steering of a female toward the left (upwards) and then to the right (down-ward) as the active speaker changes. **(B)** One loop of I1- chirps with the duration of the I1 interval indicated (top trace). The phonotactic tuning is revealed by the averaged steering velocity (mm/s) toward each type of I1-chirp over 200 loops presented (bottom trace). **(C)** Characteristic responses to the I1-chirps pooled from 15 females (*N* = 15, *n* = 200). For each chirp pattern the SEM of the response is given. **(D–F)** The lateral deviation, steering velocity, and averaged response to I2-chirps (*N* = 15, *n* = 200), using the same procedures as for the I1-chirps.

Corresponding to behavioral tests (Hedwig and Sarmiento-Ponce, 2017), two acoustic test paradigms were used based on chirps with 3 sound pulses and two pulse intervals (labeled I1 and I2) (Figures 1A,B). In the *I1-test*, the first interval (I1) of chirps was systematically set to either 5, 10, 20, 25, 30, 40, 50, and 80 ms, while keeping the duration of all pulses and the second interval (I2) at 20 ms. For example, in the I1–5 pattern, interval I1 was 5 ms whereas all pulses and I2 were 20 ms (Figure 1B, I1-test). In the *I2-test*, the second interval (I2) was systematically adjusted to 5, 10, 20, 25, 30, 40, 50, and 80 ms while the duration of the pulses and the first interval (I1) was 20 ms, (Figure 1E, I2-test). Although the chirp duration changed the chirp periods was kept at 500 ms, and chirp patterns were presented in a fixed sequence. Chirps with pulses and intervals of 20 ms are referred to as a “normal chirp,” as they correspond to natural chirps of *G. bimaculatus* (Verburgt et al., 2011).

### Behavioral tests

For the I1-test and the I2-test all 8 chirps with different intervals were combined to one “loop” lasting 4 s, sequences of 10 loops lasting 40 s in total were presented alternatingly from the left and right side (Figures 1A,B). This was repeated 10 times, so that each chirp pattern was presented 100 times from the left and 100 times from the right-hand side. Alternating stimulus paradigms were used to eliminate any lateral bias the animals may have when responding to the acoustic test patterns. To reveal the tuning of the auditory steering behavior we averaged the lateral steering velocity to chirps of the same type as a measure of the auditory evoked motor response (Hedwig and Poulet, 2005) and pooled the data for the left and right responses. Otherwise, the same set-up was used as in Hedwig and Sarmiento-Ponce (2017).

We used the looped presentation of the chirp patterns to analyze the neural responses of auditory neurons, to allow a proper comparison of the neuronal and the behavioral data.

### Intracellular recordings and staining of the neurons

The head of a cricket was fixed facing forward in a modified 2 ml Eppendorf tube using beeswax. The brain was exposed and covered by insect saline; composition in mmol/L: NaCl 140; KCl 10; CaCl_2_ 7; NaHCO_3_ 8; MgCl_2_ 1; TES 5; D-trehalose dehydrate 4, adjusted to pH 7.4. A stainless-steel platform with an embedded optic fiber was placed under the dorsal side of the brain for support and illumination. The platform also served as a reference electrode for intracellular recordings. A tungsten ring was gently placed on the ventral side of the brain to stabilize its position.

Microelectrodes were pulled from borosilicate glass capillaries (Harvard Apparatus Ltd., UK; 1 mm OD, 0.58 mm ID) using a DMZ-Universal micropipette puller (Zeitz Instruments, Martinsried, Germany). Microelectrodes were filled with 2 M potassium acetate providing resistances of 40–60 MΩ. For *in vivo* staining, the tips of the electrodes were filled with 5% Lucifer yellow CH (Sigma-Aldrich) dissolved in 0.2 M lithium chloride. The shaft was backfilled with 0.5 M lithium chloride, giving a resistance of 80–120 MΩ. The position of the microelectrode was controlled by a Leitz micromanipulator (model M; Leica Microsystems, Wetzlar, Germany). Electrode depth was monitored with a digital depth indicator (Digimatic, ID-C125MB; Mitutoyo Corporation, Japan). Intracellular recordings lasted from 1 min to more than 60 min. Recorded signals were amplified by a DC amplifier (BA-01X, NPI Electronic, Germany). Sound pulses of 250 ms duration at a carrier frequency of 4.8 kHz were used as “search” pulses to evoke field potentials and activate auditory neurons.

Auditory interneurons were recorded in the ring-like auditory neuropil in the protocerebrum (Kostarakos and Hedwig, 2012). For anatomical identification, 5% Lucifer yellow CH (Sigma Aldrich) dissolved in ddH_2_O, or 0.5% Alexa 568 hydrazide (Invitrogen) dissolved in 0.2 M lithium chloride (LiCl) was iontophoretically injected into the neurons for 2–20 min by hyperpolarizing current injection (1.5–3 nA). The brain was then dissected and fixed in 4% paraformaldehyde, dehydrated in a series of ethanol at 70, 90, 95, and 100%, and cleared in methyl salicylate. The morphology of stained neurons was examined using either a Zeiss Axiophot epifluorescence microscope (Axiophot, Carl Zeiss, Germany) with Zeiss filter sets 63 HE attached with a digital SLR camera (Canon EOS 350D; Canon) or a confocal microscope (Leica SP5, Wetzlar, Germany). Neurons were identified according to their morphology and response patterns (Kostarakos and Hedwig, 2012; Schöneich et al., 2015), and show similarities to local BNC1d and BNC2b neurons (Schildberger, 1984; Schildberger et al., 1989). For consistency we follow our previous nomenclature (Schöneich et al., 2015).

### Data recording and analysis

All recording channels (intracellular recordings, sound, current and trackball) were sampled at 20 kHz and 16-bit amplitude resolution using a CED 1401 data acquisition interface (Micro1401 mk II, CED, Cambridge, UK). Neural recordings were displayed on a computer screen using Spike 2 software (Cambridge Electronic Design) and were monitored using headphones. Data were saved to the hard disc of a PC for off-line analysis. Recorded data were analyzed with Neurolab software (Knepper and Hedwig, 1997), Spike 2 (Cambridge Electronics Design, Cambridge, UK), Prism (GraphPad Software) and Excel (Microsoft).

The number of each neuron type recorded is given by (N) and the number of stimulus repeats by (n). Characteristic responses to normal chirps were obtained and analyzed for: AN1 (*N* = 5, *n* = 10), LN2 (*N* = 2, *n* = 10), LN5 (*N* = 4, *n* = 10), LN3 (*N* = 5, *n* = 10) and LN4 (*N* = 5, *n* = 5). For the I1- and I2-test data were obtained for AN1 (*N* = 5, *n* = 5), LN2 (*N* = 2, *n* = 2), LN5 (*N* = 4, *n* = 18), LN3 (*N* = 5, *n* = 13) and LN4 (*N* = 5, *n* = 5).

We used the same looped presentation of the chirp patterns as in the behavioral tests to reveal the responses of auditory neurons. To analyze the neural activity, the number of action potential (AP) per chirp generated by the four spiking neurons of the circuit AN1, LN2, LN3, and LN4 were calculated, together with the standard error of the mean for each neuron.

For the non-spiking neuron LN5, the difference between the resting potential and the peak of a rebound was measured and the standard error of the mean was calculated for each PIR amplitude. Due to the sequential signal processing in the network the first PIR occurs after the second sound pulse, and the second and third PIR occur after the third sound pulse. Therefore, the latency of the first PIR is measured in relation to the second pulse and the latency of the subsequent PIRs is measured relative to the third pulse.

Neural responses to test patterns were analyzed by Repeated Measure ANOVA with Tukey’s *post-hoc* test if the data were parametric, and Friedman test with Dunn’s multiple comparisons test if the data were non-parametric. Results with a “*p*” value less than 0.05 are considered as significant. Pearson’s correlation coefficient was calculated to correlate the amplitude of the PIR and the following inhibition.

## Results

### Behavioral responses

When presented with looped chirps of the I1 or I2-test patterns the crickets showed clear steering responses toward the acoustic signal. They changed the walking direction whenever the stimulus side was altered, as indicated by the lateral deviation toward the active speaker (Figures 1A,D). The lateral deviation does not directly reveal the specific steering responses to the 8 different types of chirps combined in a loop. For each animal we averaged the steering velocity over the time course of the 200 loops presented in the I1- and I2-tests and measured the amplitude of the change in steering velocity initiated by each type of chirp as an indicator of the phonotactic response (Figures 1B,E). Changes in steering velocity toward a chirp occurred after 60.33 ± 4.3 ms and reached a peak after 200.03 ± 4.2 ms indicating that already the first two sound pulses of a chirp triggered the auditory response and that the response lasted for the duration of the chirp. We did not consider any further steering reactions to subsequent sound pulses in a chirp in the time course of the response, see e.g., I2–80.

Pooling of the data over all females tested (I1-test: *N* = 15; I2-test: *N* = 15) revealed a maximum auditory steering response at the normal chirp pattern and rather broad phonotactic tuning curves, with consistent steering even to chirps with short (e.g., I1–5 and I2–5) and long intervals (e.g., I1–80 and I2–80) (Figures 1C,F). These tuning curves were used as reference for the neuronal data.

### Neuronal activity

**FIGURE 2:**
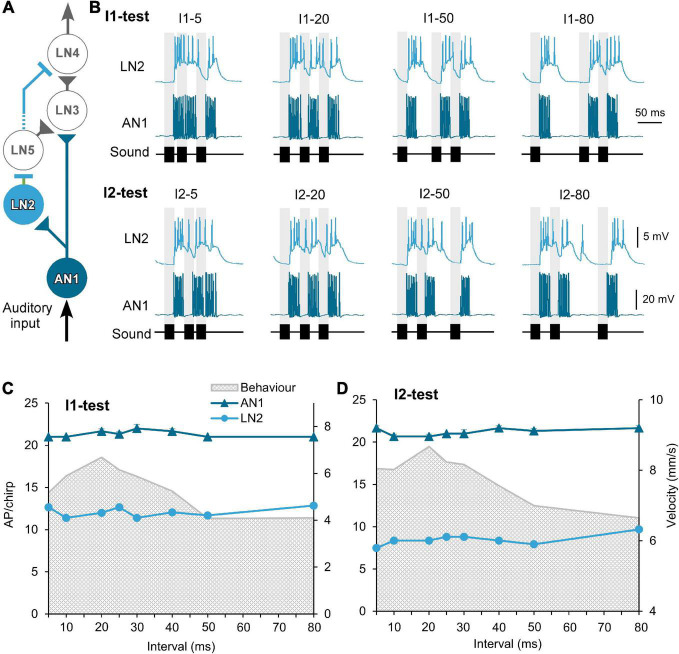
Response of the ascending neuron AN1 and the inhibitory neuron LN2 to the I1- and I2-tests. **(A)** The delay-line and coincidence detector circuit with AN1 (dark blue) and LN2 (light blue), triangles indicate excitatory and lines inhibitory connections. **(B)** Activity of AN1 and LN2 in response to four example I1- or I2-chirps, labeled above each response. Gray bars indicate the timing of sound pulses. **(C,D)** Tuning of AN1 and LN2 activity (AP/chirp) in response to the I1-test and the I2-test. The tuning of phonotactic behavior is indicated by gray shade. AN1 activity was averaged from 5 animals (*N* = 5, *n* = 5). LN2 activity was averaged from 2 animals (*N* = 2, *n* = 2). Diagram in **(A)** modified from Schöneich et al. (2015).

**FIGURE 3:**
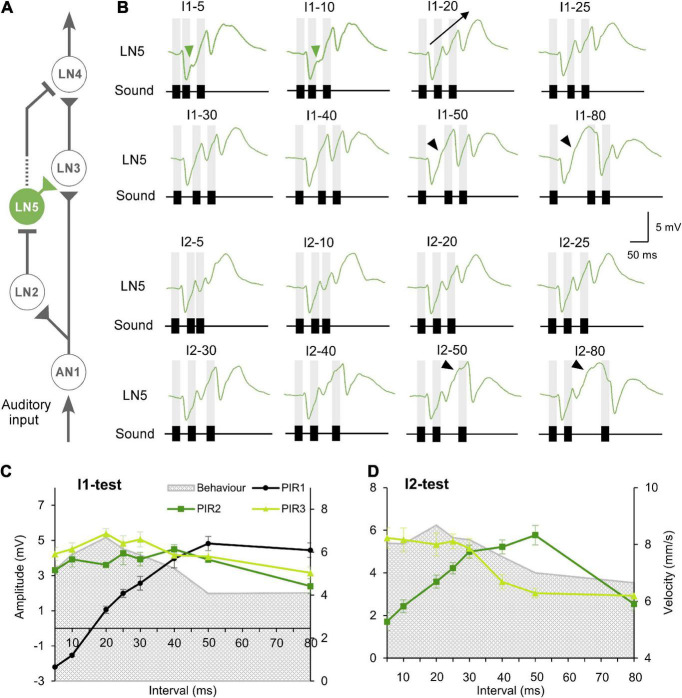
Responses of the non-spiking neuron LN5 to the I1- and I2-tests. **(A)** The delay-line and coincidence detector circuit with LN5 (green). **(B)** Averaged response of LN5 to the I1- and I2-tests (*N* = 4, *n* = 18). Chirps are labeled above each response; gray bars indicate the timing of sound pulses. A black arrow indicates the increase in membrane potential over successive rebounds in response to I1–20. Green arrowheads indicate the deflection in membrane potential during rebounds over short intervals and black arrowheads mark deflections over long time intervals. **(C,D)** Maximum amplitude of the three rebounds (PIR1 to PIR3) over the I1-test and the I2-test, error bars indicate SEM, phonotactic tuning is indicated by gray shade.

We recorded the activity of auditory brain neurons in the ventral protocerebrum (Kostarakos and Hedwig, 2012) in response to chirps with systematically varied pulse intervals analyzing the flow of activity in the delay-line coincidence detector circuit as depicted in Figures 2A, 3A, 6A.

**FIGURE 4:**
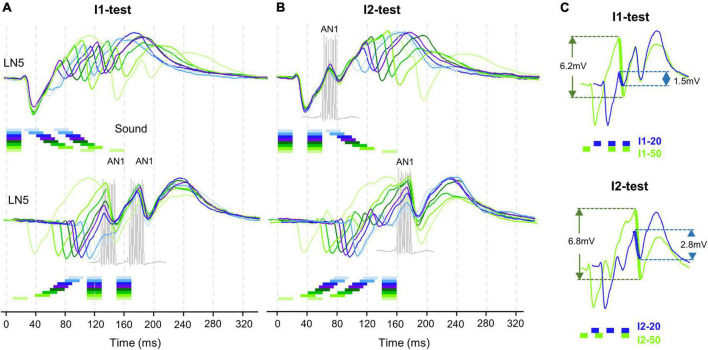
Timing of LN5 activity elicited by I1- and I2-test. **(A)** Averaged LN5 membrane potential in response to chirps of the I1-test aligned to the onset of the first (top) and second (bottom) sound pulse (*N* = 4, *n* = 18). **(B)** Averaged LN5 membrane potential in response to chirps of the I2-test aligned to the onset of the first (top) and the third (bottom) sound pulse. For test intervals smaller than 30 ms, the chirps and responses are colored in light to dark blue and purple, for intervals equal to or larger than 30 ms, they are colored as dark to light green, respectively. The timing and duration of AN1 activity elicited by the second and the third sound pulses is indicated with example responses, in gray. **(C)** Comparison of inhibition amplitude caused by pulse 2 for the response to I1–20 and I1–50, and of the inhibition caused by pulse 3 for I2–20 and I2–50. LN5 response to I1–20 and I2–20 in blue and to I1–50 and I2–50 indicated in green, relevant potential changes are indicated by thick lines.

**FIGURE 5:**
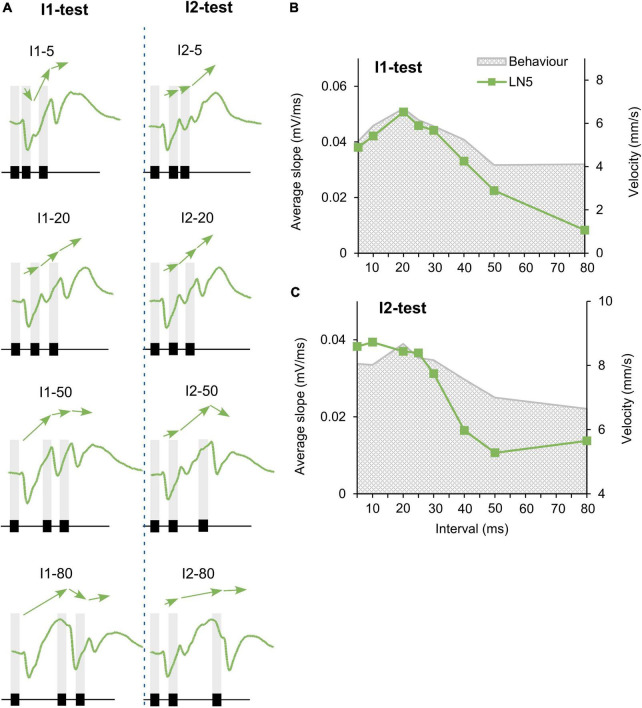
Change in PIR amplitude over the time course of chirps. **(A)** Maximum amplitude of subsequent rebounds in response to four example I1-chirps and I2-chirps, (*N* = 4, *n* = 18). Arrows indicate the slope between adjacent rebound peaks. Chirps tested are indicated above each recording, gray bars indicate the timing of sound pulses. **(B,C)** The tuning of the mean “slope” of LN5 for the I1-test and I2-test, as averaged from slope 1, slope 2, and slope 3 (*N* = 4, *n* = 18). Gray shade indicates phonotactic response.

**FIGURE 6:**
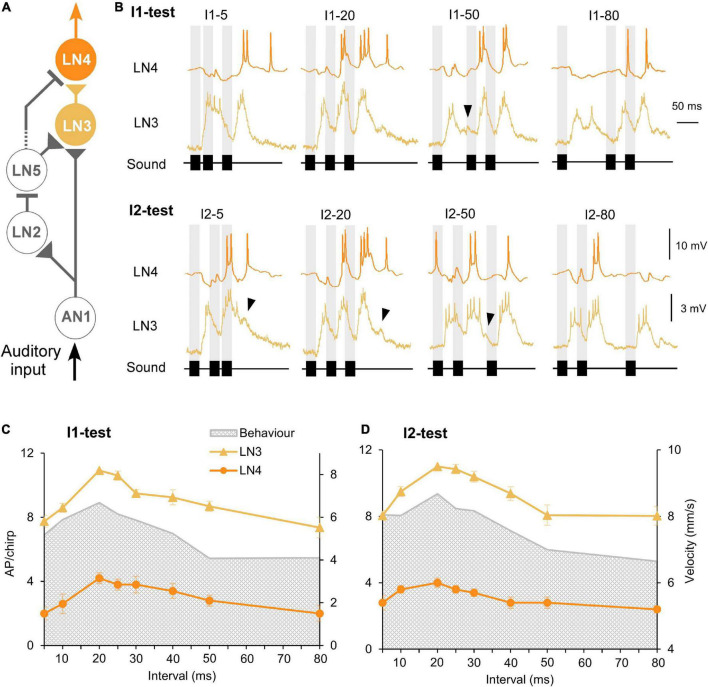
Response of the coincidence detector neuron LN3 and the feature detector neuron LN4 to I1- and I2-tests. **(A)** The delay-line and coincidence detector circuit with LN3 (yellow) and LN4 (orange), triangles indicate excitatory and lines inhibitory connections. **(B)** Activity of LN3 and LN4 in response to I1- or I2-chirps, labeled above each response. Gray bars indicate the timing of sound pulses, and arrowheads indicate a delayed depolarization following the first or the second burst of spikes. **(C,D)** Tuning of LN3 (*N* = 5, *n* = 13) and LN4 (*N* = 5, *n* = 5) activity (AP/chirp) in response to the I1-test and the I2-test, error bars represent SEM, gray shade indicates phonotactic response.

#### Auditory neurons with no temporal selectivity: Response of the ascending neuron AN1 and of the local neuron LN2

Spike activity of the ascending neuron AN1 is driven by the afferent activity and copies and forward the temporal pattern of 4.8 kHz sound stimuli to the brain (Wohlers and Huber, 1982; Schildberger, 1984; Kostarakos and Hedwig, 2012; Figures 2A,B). The neuron responded to 20 ms pulses with 7.3 ± 0.3 AP/pulse, after a latency of 18.9 ms and its spike activity lasted for 22.1 ± 0.2 ms (*n* = 10). In response to chirps with short I1 or I2 intervals (I1–5 or I2–5), the 5 ms intervals between the sound pulses were not obvious in its spike pattern (Figure 2B). With longer intervals AN1 reliably copied the temporal pattern of the chirps (Wohlers and Huber, 1982; Schildberger, 1984; Schildberger et al., 1989; Kostarakos and Hedwig, 2012). The number of spikes elicited by chirps of the I1- and I2-tests were very similar across the different chirps and ranged from 20.7 ± 0.5 to 22.0 ± 0.5 AP/chirp (Table 1) and AN1 spiking response did not represent the tuning of the phonotactic behavior (Figures 2C,D) (I1: *p* = 0.1526, I2: *p* = 0.2918, Friedman test, *N* = 5, *n* = 5).

**TABLE 1:** Number of the spikes of the four spiking neurons in response to a pulse and chirps.

Neuron	AN1	LN2	LN3	LN4
**Chirp**				
20 ms pulse	6.5 ± 0.0	5.3 ± 0.1	2.8 ± 0.2	IPSP
I1–5	21 ± 0.0	12.7	7.7 ± 0.4	2 ± 0.4
I1–10	21 ± 0.0	11.4	8.6 ± 0.3	2.6 ± 0.6
I1–20	21.7 ± 0.3	12	10.9 ± 0.0	4.2 ± 0.3
I1–25	21.3 ± 0.3	12.7	10.6 ± 0.3	3.8 ± 0.3
I1–30	22 ± 0.5	11.4	9.5 ± 0.2	3.8 ± 0.5
I1–40	21.7 ± 0.3	12.1	9.2 ± 0.5	3.4 ± 0.5
I1–50	21 ± 0.0	11.7	8.7 ± 0.3	2.8 ± 0.3
I1–80	21 ± 0.0	12.8	7.4 ± 0.6	2 ± 0.5
I2–5	21.7 ± 0.3	7.5	8.1 ± 0.3	2.8 ± 0.4
I2–10	20.7 ± 0.3	8.4	9.5 ± 0.3	3.6 ± 0.2
I2–20	20.7 ± 0.3	8.4	11 ± 0.0	4 ± 0.3
I2–25	21 ± 0.0	8.8	10.8 ± 0.3	3.6 ± 0.2
I2–30	21 ± 0.5	8.8	10.4 ± 0.3	3.4 ± 0.2
I2–40	21.7 ± 0.3	8.4	9.4 ± 0.4	2.8 ± 0.3
I2–50	21.3 ± 0.3	8	8.1 ± 0.6	2.8 ± 0.3
I2–80	21.7 ± 0.3	9.7	8.0 ± 0.6	2.4 ± 0.2

LN4 responds to a single sound pulse with an IPSP. Responses are given with SEM.

The inhibitory local brain neuron LN2 is proposed to be driven by AN1 (Figure 2A; Schöneich et al., 2015). In response to a 20 ms pulse LN2 depolarized with a latency of 19.9 ± 0.2 ms, it started spiking after 22.2 ± 0.2 ms for 25.0 ± 1.9 ms, while the underlying depolarization lasted for 58.4 ± 3.8 ms and declined only slowly (Table 2). Therefore, when LN2 was stimulated with chirps containing short I1- or I2-intervals LN2′s spike activity occurred on a plateau-like depolarization. Short pulse intervals were not represented in its spike pattern. Even for medium I1 or I2 intervals of 20 and 30 ms, the first and the second sound pulses elicited a depolarization, which did not fully return to the resting membrane potential; only when exposed to chirps with long intervals between 40 and 80 ms did the membrane potential repolarize (Figure 2B).

**TABLE 2:** Response latency of the five neurons.

Neuron	Latency/duration	Normal chirp			Single pulse
		1st pulse	2nd pulse	3rd pulse	20 ms
**AN1**	LAT_*spike*_	18.9 ± 0.1	18-^# ± 0.2	19.6 ± 0.1	
	DURA_*spike*_	22.1 ± 0.2			
**LN2**	LAT_*depo*_	19.9 ± 0.2	21.7 ± 0.5	22.5 ± 0.5	
	LAT_*spike*_	22.2 ± 0.2	24.5 ± 0.5	26.4 ± 0.5	
	DURA_*depo*_	39.2 ± 0.7	40.6 ± 0.6	54.9 ± 1.9	58.4 ± 3.8
	DURA_*spike*_	25.0 ± 1.0	20.5 ± 1.7	10.0 ± 0.7	25.0 ± 1.9
	DURA_*initial*_				10.3 ± 0.2
**LN5**	LAT_*inh*_	26.4 ± 0.2	25.7 ± 0.5	26.2 ± 0.4	
	LAT_*maxinh*_	36.4 ± 0.5	35.4 ± 1.0	36.9 ± 0.3	
	LAT_*peak*_				89.4 ± 2.2
	DURA_*PIR*_				177.9 ± 1.8
**LN3**	LAT_*depo*_	23.2 ± 0.4	23.4 ± 0.4	24.0 ± 0.2	
	LAT_*spike*_	34.1 ± 0.8	27.3 ± 0.4	29.8 ± 0.5	
	DURA_*depo*_	33.8 ± 0.3	33.7 ± 1.3	41.5 ± 2.1	54.0 ± 0.3
	DURA_*spike*_	6.7 ± 0.6	16.7 ± 1.3	14.2 ± 0.8	8.7 ± 0.1
**LN4**	LAT_*inh/spike*_	24.9 ± 0.3	30.3 ± 0.5	29.1 ± 0.2	
	DURA_*depo*_		19.3 ± 0.5	30.6 ± 2.0	
	DURA_*spike*_		9.7 ± 1.4	16.5 ± 1.1	
	DURA_*inh*_				75.6 ± 1.2

LAT*_depo_*: the latency of the depolarization to each sound pulse. LAT*_spike_*: the latency of the spikes to each sound pulse. LAT*_inh_*: the latency of the start of the inhibition elicited by each sound pulse. LAT*_maxinh_*: the latency of the maximum of the inhibition elicited by each sound pulse. LAT*_peak_*: Latency of peak of the post-inhibitory rebound elicited by a single 20 ms pulse. LAT*_inh/spike_*: latency of the initial inhibition in LN4 elicited by the first sound pulse and the latency of the spikes elicited by the second pulse or the third pulse.

DURA*_depo_*: the duration of the depolarization elicited by each sound pulse. DURA*_spikes_*: the duration of the spiking activity elicited by each sound pulse. DURA*_initial_*: the duration of the initial intense spikes elicited by a single 20 ms pulse. DURA*_PIR_*: the duration of the post-inhibitory rebound elicited by a single 20 ms pulse. DURA*_inh_*: the duration of the inhibition elicited by a single 20 ms pulse in LN4.

LN2 responded to the first sound pulse of a chirp with phasic spike activity, reaching spike rates of 236.15 ± 6.48 Hz for the initial 3 spikes, this transient activity lasted 10.3 ± 0.2 ms, with an average of 5.3 ± 0.1 AP per pulse. Spike activity to the subsequent sound pulses was considerably lower and was only 2.4 ± 0.1 AP in response to the third pulse of a normal chirp. Over all chirps tested the level of activity of LN2 remained similar across the I1- or I2-test; it showed only a difference of 1.4 spikes in I1-test and 2.2 spikes in I2-test (I1–10: 11.4 AP/chirp and I1–80: 12.8 AP/chirp; I2–5: 7.48 AP/chirp and I2–80: 9.68 AP/chirp), (Figures 2C,D). The resulting tuning curves of LN2 do not reflect the phonotactic behavior, indicating no temporal selectivity in its response. Compared to the AN1 activity, LN2 responded to the I1- and I2-pattern with 43.2 ± 1.1% and 60.05 ± 1.04% fewer spikes (I1-test: AN1 21.3 ± 0.1 AP/chirp, LN2 12.1 ± 0.2 AP/chirp; I2-test: AN1 21.2 ± 0.1 AP/chirp, LN2: 8.5 ± 0.2 AP/chirp).

#### Response dynamics of the non-spiking delay-line neuron LN5

The non-spiking brain neuron LN5 plays a key role in generating a delayed excitation matching the timing of the species-specific pulse pattern (Schöneich et al., 2015). Its membrane potential is driven by the inhibitory input from LN2 (Figure 3A) and by its intrinsic properties. In response to a single 20 ms sound pulse LN5 generated an inhibition followed by a pronounced delayed PIR. The inhibition started after 26.4 ± 0.2 ms and reached a maximum of −4.4 ± 0.1 mV at 36.4 ± 0.5 ms. Normal chirps elicited a rhythmic membrane potential oscillation driven by the pulse pattern with a typical sequence of inhibition and PIR depolarization (Figure 3B, I1–20 and I2–20).

When exposed to I1–5 or I1–10 (Figure 3B) LN5 generated the initial inhibition in response to the first sound pulse, but the following PIR was not fully developed, and resulted in only a small deviation in the depolarizing membrane potential (Figure 3B, green arrowhead). At these short pulse intervals AN1 and LN2 do not copy the pulse pattern (Wohlers and Huber, 1982; Kostarakos and Hedwig, 2012; Schöneich et al., 2015), and there was only a minor inhibitory deflection in the LN5 response in response to the second sound pulse. The third sound pulse elicited an inhibition which defined the peak of the second PIR and subsequently initiated a pronounced third PIR, which however was not terminated by a subsequent inhibition. In response to I1–20 and I1–30 chirps (Figure 3B), each of the three sound pulses elicited a separate PIR with a peak above the resting potential and the amplitude of the three PIRs gradually increased over the time course of the chirps (Figure 3B, I1–20, black arrow).

For long I1 or I2-intervals of 80 ms, the PIR developed from the peak of the preceding inhibition over a time course of 67.4–72.0 ms and reached a broad maximum. In the rising phase of the first and second rebound a deflection of the membrane potential occurred (Figure 3B, black arrowheads), which was not linked to a sound pulse.

To quantify the LN5 activity we measured the amplitude of the three PIRs relative to the resting membrane potential and plotted these together with the behavioral response over the loop of tested intervals (Figures 3C,D and Table 3). In the I1-test the first PIR showed the strongest gain, as with increasing duration of I1 the rebound had more time to develop; it increased to a maximum of 4.8 ± 0.4 mV at I1–50 and then decreased to 4.4 ± 0.4 mV at I1–80 (Table 3). The amplitude of the second PIR showed overall a rather flat course with 4–5 mV for I1–5 to I1–40 and then declined to 2–3 mV at I1–80 (Figure 3C), while the third rebound tuning curve followed the behavior with a peak of 5.4 ± 0.3 mV at I1–20 and a gradual decline toward I1–80. In comparison, only the tuning curve for the third rebound indicated a match with the behavior. In the I2-test (Figure 3D) the amplitude of the first PIR was not affected, it was 1.9 ± 0.2 mV throughout and is not shown in the graph. The amplitude of the second rebound increased from 1.7 ± 0.4 mV to 5.8 ± 0.56 mV as I2 increased from 5 with to 50 ms, and it declined to 2.5 ± 0.3 mV with I2 reaching 80 ms. The amplitude of the third PIR was between 5.5 and 5.2 mV when I2 increased from 5 to 25 ms, it then dropped to 3.1 ± 0.2 mV at I2–50 and remained at a similarly low level for I2–80. In this way the tuning of the third rebound was different and qualitatively matched the phonotactic tuning for the I2-test.

**TABLE 3:** Membrane potential (mV) of the PIRs of LN5.

PIRs	PIR1	PIR2	PIR3
**Chirp**			
I1–5	−2.2 ± 0.0	3.3 ± 0.2	4.2 ± 0.4
I1–10	−1.5 ± 0.1	3.9 ± 0.4	4.5 ± 0.3
I1–20	1.1 ± 0.2	3.6 ± 0.1	5.4 ± 0.3
I1–25	2.0 ± 0.2	4.3 ± 0.6	4.8 ± 0.4
I1–30	2.6 ± 0.4	4.0 ± 0.4	5.1 ± 0.4
I1–40	4.0 ± 0.5	4.5 ± 0.3	4.2 ± 0.3
I1–50	4.8 ± 0.4	4.0 ± 0.2	4.1 ± 0.3
I1–80	4.4 ± 0.4	2.4 ± 0.4	3.2 ± 0.3
I2–5	1.9 ± 0.2	1.7 ± 0.4	5.6 ± 0.5
I2–10		2.4 ± 0.3	5.6 ± 0.5
I2–20		3.6 ± 0.3	5.3 ± 0.5
I2–25		4.2 ± 0.3	5.5 ± 0.3
I2–30		5.0 ± 0.3	5.2 ± 0.4
I2–40		5.2 ± 0.3	3.6 ± 0.3
I2–50		5.8 ± 0.5	3.1 ± 0.2
I2–80		2.5 ± 0.3	3.0 ± 0.1

Values are given in mV with SEM.

#### LN5 post-inhibitory rebounds and timing of AN1 activity

To further reveal the time course of the LN5 rebound activity, we superimposed the PIRs in respect to the first (Figures 4A,B top) or last (Figures 4A,B bottom) sound pulse of the I1- and I2-chirps. The superpositions aligned to the first sound pulse of the chirps demonstrate that the time course of the first inhibition is constant for all I1-test patterns, while the rising phase and timing of the rebound depends on the duration of the I1-interval. Based on the delay-line and coincidence detector model, the response of the coincidence detector LN3 is driven by the LN5 membrane potential and AN1 spike activity. For both I1- and I2-tests based on the latency (18.9 ± 0.1 ms) and the response duration (22.1 ± 0.2 ms) of AN1 we highlight the timing of AN1 activity elicited by the second and the third pulses in Figure 4 as they are relevant for the coincidence detection process (Figures 4A,B, grey). Corresponding to the interval between the start of the AN1 spiking response and the start of the LN5 inhibition (latency 26.4 ms) the AN1 response to the second sound pulse overlapped with the rising phase of the first rebound, and the AN1 response to the third pulse overlapped with the rising phase of second rebound for about 7 ms; corresponding to the first 2–3 spikes of the AN1 response. This time of coincidence is determined by the flow of activity in the network and the latencies of the neurons involved.

#### The interplay of inhibition and rebound of LN5

The interplay of inhibition and rebound depolarization may give insight into the driving forces for the LN5 membrane potential changes. When the second or the third sound pulse triggered an inhibition at a high rebound amplitude this caused a stronger inhibition than when occurring at a low rebound amplitude (Figures 4A,B). For example, for I1–50 the membrane potential of the first rebound reached an amplitude 4.8 ± 0.4 mV while for I1–20 it was 1.1 ± 0.2 mV; in case of I1–50 the inhibition reduced the membrane potential of the first rebound by 6.2 ± 0.2 mV while for I1–20 the drop was only 1.4 ± 0.1 mV (Figure 4C, I1-test). The same interplay occurred for PIRs in I2-test (Figure 4C, I2-test). The amplitude of the inhibition caused by the third sound pulse was 6.8 ± 0.5 mV for I2–50 and only 2.8 ± 0.3 mV for I2–20. Moreover, in both examples the inhibition reached a lower membrane potential when imposed on higher rebound amplitudes than with the smaller rebound amplitudes.

Overall, within the first 30 ms of a developing rebound, the inhibition in response to the next sound pulse caused only a small reduction in membrane potential of 0.3 to 1.4 mV, whereas at a later stage with a higher rebound potential at I1–80 and I2–80 the effect of the inhibition was considerably stronger, reducing the membrane potential by up to 7.1 mV. Thus, the maximum amplitude of the resulting inhibition was positively correlated to the maximum amplitude of the preceding rebound with a correlation coefficient of *R*^2^ = 0.98. The recovery of the phasic LN2 activity after long intervals and a decrease in the driving force for the rebounds might contribute to this phenomenon.

#### The post-inhibitory rebound activity of the non-spiking neuron shows selectivity to the species-specific pulse pattern

Our recordings demonstrated an overall increase of the LN5 membrane potential over the time course of the I1- and I2-chirps, most obvious in response to the normal chirps (Figure 5A, I1–20 and I2–20, arrows), with the subsequent PIRs increasing in amplitude. Chirps with short or long pulse intervals elicited different changes in the membrane potential (e.g., Figure 5A I1–5, I1–50, I1–80). In the same way we demonstrate the change in rebound maxima for the I2-test (Figure 5A, right). For both test paradigms we analyzed and plotted the mean membrane potential change over the interval duration of the test patterns, which is called the “slope” here.

For a complete LN5 response to a chirp, slope 1 is calculated by the difference between the resting membrane potential and the peak of the first rebound over the time from the onset of the inhibition to the peak of the first rebound. Slope 2 is the amplitude difference between the peaks of the second and the first rebounds over the time interval between the two peaks and slope 3 is the difference between the peaks of the third and the second rebounds over the interval between these two peaks. The overall LN5 response to a particular chirp was calculated as the mean of the slopes.

For the I1-test, the tuning curve increases from I1–5 to a peak at I1–20, and it then gradually declines toward I1–80 (Figure 5B). For the I2-test, the tuning curve was at a high level for I2–5 to I2–25 ms, it then decreased to I2–50 and stayed at a low level until I2–80 (Figure 5C). Both tuning curves reveal that–similar to the tuning of the third rebound (see Figures 3C,D)–the gradual increase of the depolarization that shapes the response of LN5 over the time course of the chirps matches the behavioral tuning (Figures 5B,C). This indicates that LN5 may not only function as a delay-line but that its membrane potential change over a chirp is also tuned to the temporal pattern of the species-specific sound pulses.

#### Auditory brain neurons with temporal selectivity: Response of the coincidence detector LN3 and the feature detector LN4

The coincidence detector neuron LN3 receives a direct input by AN1 spikes and a graded input from the non-spiking delay-line neuron LN5 (Figure 6A); when their depolarizing activity coincides it boosts the LN3 response to a sound pulse (Schöneich et al., 2015). For the first and last sound pulse of a chirp AN1 and LN5 rebound activity do not overlap: in response to the first pulse LN3 is only driven by AN1 spike activity and after the last sound pulse it is only driven by the LN5 PIR. Thus, for these pulses the delay-line coincidence detector is not fully activated, which needs to be considered in respect to the timing of phonotactic steering motor activity. Only by the second and the third pulse is the coincidence detector fully activated at the neuronal level.

When exposed to chirps with short intervals (I1–5, I2–5) the LN3 neuron generated a prolonged depolarization in response to the two adjacent pulses which did not reveal the 5 ms interval and reflected the corresponding response pattern of AN1 and LN2. When exposed to medium I1 and I2 intervals (20–30 ms) LN3 generated three separated depolarizations with bursts of spikes; the response to the second pulse was significantly stronger than the response to the first pulse (*P* < 0.0001, paired *t*-test), this effect however vanished with increasing interval durations. At long intervals (40–80 ms), a subthreshold depolarization sometimes occurred with a latency of 77.8 ± 2.0 ms while the membrane potential decreased following a burst of LN3 spikes (Figure 6B, black arrowheads), this depolarization likely indicates the graded input from LN5.

Besides the spiking response to the pulse pattern, the membrane potential of LN3 also revealed an overall gradual depolarization for normal chirps. If the non-spiking LN5 neuron drives the activity of LN3 with a graded transmitter release, the overall depolarization of LN3 should be linked to the increased depolarization of LN5 occurring at normal chirps. As the I1-interval increased, it could be expected that the response of LN3 should become stronger since an increased rebound potential of LN5 will coincide with AN1 spike activity (see Figure 4), such an effect, however, was not observed.

Quantitative analysis of the number of spikes/chirp over the test patterns showed that for both interval tests, the tuning of LN3 activity matched the tuning of the phonotactic behavior, showing a peak at the best phonotactic response and a gradual declining activity toward short and long pulse intervals (Figures 6C,D). For the I1-test, LN3 activity significantly increased from 7.7 ± 0.4 to 10.9 ± 0.0 AP/chirp as interval I1 increased from 5 ms to 20 ms (I1–5 vs. I1–20: *p* < 0.0001; I1–10 vs. I1–20: *p* = 0.0007, Friedman test, *N* = 5, *n* = 13), the activity then significantly decreased to 7.4 ± 0.6 AP/chirp at I1–80 ms (I1–80 vs. I1–20: *p* < 0.0001, Friedman test, *N* = 5, *n* = 13). The same trend was observed in the I2-test: LN3 activity significantly increased from 8.1 ± 0.3 to 11 ± 0.0 AP/chirp as interval I2 was extended from 5 to 20 ms (I2–5 vs. I2–20: *p* < 0.0001; I2–10 vs. I2–20: *p* = 0.0285, Friedman test, Tukey’s, *N* = 5, *n* = 13) and then decreased to 8.0 ± 0.6 AP/chirp at I2–80 ms (I2–80 vs. I2–20: *p* = 0.0003, Friedman test, Tukey’s, *N* = 5, *n* = 13), again similar to the behavioral tuning. The mean LN3 spike activity in response to the I1- and I2-pattern, was 57.51 ± 1.79% and 55.70 ± 2.11% lower than the activity of AN1.

The feature detector LN4 receives excitatory input from LN3 and is inhibited by from neuron LN2 (Figure 6A; Schöneich et al., 2015). In the I1-test, the initial response of LN4 to chirps with I1–5 was dominated by a pronounced inhibition, in which the inhibitory response to the first two sound pulses merged and only the third pulse generated 2 ± 0.4 AP (Figure 6B, I1-test). For medium I1-interval durations (20 to 30 ms), LN4 generated an inhibition in response to the first sound pulse, followed by two pronounced depolarizations with 2–3 spikes elicited by the second pulse and 3–4 spikes by the third pulse. As interval I1 increased further (I1–40 to I1–80), the initial inhibition dominated the LN4 activity and was followed by 1–2 spikes in response to the second and third pulse.

When exposed to I2-chirps with short intervals (I2–5 and I2–10), LN4 activity started with an inhibition, while the subsequent response to the sound pulses separated by a short interval merged, leading to one short burst of spikes (Figure 6B, I2-test). The duration of the underlying depolarization was only 22.0 ± 0.7 ms and did not represent the 45 ms duration of both sound pulses (each 20 ms) and the short interval (5 ms), (see Kostarakos and Hedwig, 2012). When stimulated with medium I2-intervals (20 to 30 ms), the second and third sound pulse elicited two bursts of spikes, with the third always initiating a longer and stronger depolarization and spike activity than the second pulse (second pulse: 18.6 ± 0.7 ms; third pulse: 29.8 ± 0.5 ms). With increasing duration of the I2-intervals (I2–40 to I2–80) the third sound pulse gradually failed to elicit a depolarization, and rather caused an inhibition again like the first pulse of a chirp (see I2–80), and LN4 generated only one burst of spikes occurring in response to the second pulse.

We compared the LN4 activity (AP/chirp) and the phonotactic behavior for the I1- and I2-test (Figures 6C,D). Plotting the LN4 spike activity over the I1-test (Figure 6C and Table 1), shows a steep increase from 2.0 ± 0.4 AP/chirp at I1–5 to its peak at with 4.0 ± 0.3 AP/chirp at I1–20 (I1–5 vs. I1–20: *p* = 0.0109, Friedman test, Tukey’s, *N* = 5, *n* = 5). The neuronal activity then gradually declines from 3.6 at I1–25 to 0.8 ± 0.2 AP/chirp at I1–80 (I1–80 vs. I1–20: *p* = 0.0169, Friedman test, *N* = 5, *n* = 5) (Figure 6C). For the I2-test the response of LN4 increased from 2.8 ± 0.4 to 4.0 ± 0.3 AP/chirp as interval I2 increased from 5 ms to 20 ms and then gradually decreased to 2.4 ± 0.2 AP/chirp for I2–80 ms (Figure 6D). Both LN4 tuning curves match the tuning of the phonotactic behavior. The mean LN4 response over the tests was 66.24 ± 0.94% lower than LN3 activity, and 84.99 ± 0.95% lower than AN1 activity. Due to the sparse spiking activity of LN4 the maximum difference in the mean number of spikes over the tuning curves was only 2.2 (I1-test) and 1.6 (I2-test) spikes (see also Schöneich et al., 2015).

#### The sequential filtering of the five neurons to the chirps in I1- and I2-tests

**FIGURE 7:**
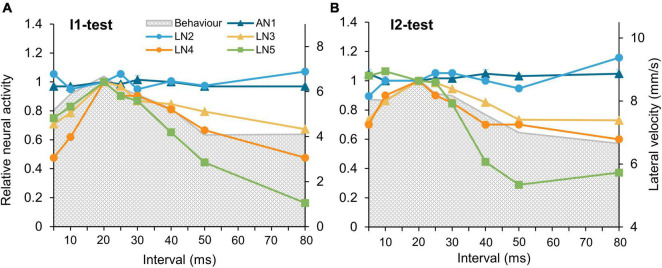
Tuning of the phonotactic behavior and the tested auditory neurons to I1-, and I2-tests. **(A,B)** Tuning curves of the five neurons in the pattern recognition circuit to the I1-test and the I2-test, tuning of phonotactic behavior is indicated by gray shade. Neural responses of the neurons to the normal chirp were set as 1 and relative responses to other chirps calculated. For clarity, the maximum phonotaxis response is aligned to the maximum neuronal response. Error bars indicate SEM.

To compare the neuronal tuning curves we normalized the activity of each neuron to its activity elicited by a normal chirp and compared all tuning curves with the tuning of the phonotactic behavior (Figures 7A,B). In both tests, AN1 and LN2 did not show any tuning to changes in interval durations of I1 or I2, whereas both LN3 and LN4 had tuning curves with a peak response at the normal chirp, and an overall match to the phonotactic behavior. Moreover also the tuning of LN5 based on the overall change in its membrane potential in response to a chirp corresponded well to the behavior (Figures 7A,B; green line) especially for intervals longer than 20 ms. A mismatch between LN5 activity and the tuning occurs for I2-intervals shorter than 20 ms. This may be a consequence of scaling or may imply that different filter and processing mechanisms are relevant in the circuit when intervals are shorter than 20 ms, as coding of short intervals is already limited at the level of AN1 spike activity. The fits of the tuning curves indicate that the neurons in the delay-line and coincidence-detector circuit robustly represent and filter the temporal information of the species-specific pulse pattern, even when presented in a looped stimulus pattern.

## Discussion

The delay-line and coincidence-detection circuitry for cricket song pattern recognition (Schöneich et al., 2015), had been tested with a paradigm in which pulse duration and pulse interval were altered at the same time. Here we varied only one specific pulse interval in the chirps to analyze the subsequent development of the PIRs and their functional role for pattern recognition and we compared the tuning of the auditory brain neurons with the tuning of the phonotactic behavior.

### Behavioral evidence

The behavioral tuning curves toward the I1- and I2-test revealed peak phonotactic responses at the normal chirp pattern, as reported in previous experiment for *G. bimaculatus* and *G. campestris* (Thorson et al., 1982; Doherty and Pires, 1987; Poulet and Hedwig, 2005; Hedwig and Sarmiento-Ponce, 2017). However, the tuning of the behavior was broad, as even chirps with single short (5 ms) and long (80 ms) intervals elicited phonotactic steering. This is different to the steering behavior reported by Hedwig and Sarmiento-Ponce (2017), who used the same stimuli but presented each type of I1 and I2-chirps for 1 min sequentially before switching to another type of chirps, to test phonotactic responses. In these tests the females showed a clear preference for the species-specific pulse interval and did not steer to chirps with one very short or long interval. This difference in the tuning may be due to the design of stimulus patterns, as only one interval in a chirp was changed and the others were kept near the optimum for phonotaxis, but the same reasoning would be valid for the previous tests (Hedwig and Sarmiento-Ponce, 2017). The difference rather may be due to a modulatory effect that occurs when females respond to the species-specific song pattern, which significantly enhances the response to non-attractive chirps and artificial amplitude modulated odd-chirps (Poulet and Hedwig, 2005; Bent and Hedwig, 2021), like the context dependent aftereffects which can broaden the tuning of phonotaxis (Doherty, 1985). Given a modulatory effect on phonotactic steering, in the looped presentation of the chirp patterns, the normal chirps with pulse intervals of 10–30 ms likely imposed such a modulation on the response to subsequent chirps with long and short intervals. The resulting tuning is therefore broader than in a standard behavioral test situation, in which only one type of chirps is sequentially presented. As we used the same auditory stimulus paradigm for the neurophysiological recordings, we could compare both sets of data and gain new insight into the neural processing underlying phonotaxis.

### No pattern selectivity at the level of the ascending pathway and local brain neuron LN2

At the level of the ascending interneuron our data confirm that AN1 copies the auditory pulse pattern to the brain without a particular temporal selectivity (Schildberger et al., 1989; Kostarakos and Hedwig, 2012). AN1 does not resolve pulses separated by short intervals, such responses merge and limit the temporal resolution for high pulse repetition rates (Wohlers and Huber, 1982; Schöneich et al., 2015).

The local interneuron LN2 followed the activity of AN1 with a latency of about 1 ms, and an initial phasic spike response over 5–10 ms. Like AN1 also LN2 activity did not resolve short pulse intervals and was not tuned to the test patterns (Kostarakos and Hedwig, 2012). Within the circuitry LN2 functions as a “sign” inverter and provides inhibitory input to the delay-line neuron LN5 and the feature detector LN4 (Schöneich et al., 2015). An inhibitory neuron with a function like LN2 was proposed by Crawford (1997) and Large and Crawford (2002) for an auditory interval selective circuit in the mesencephalon of the mormyrid fish *Pollimyrus adspersus*. In their model this neuron, causes an inhibition leading to a subsequent PIR in a coincidence detector neuron. Inhibition driving PIR for the processing of temporal patterns has also been proposed in frogs (Leary et al., 2008; Elliott et al., 2011; Rose, 2014), and mammals (Kuwada and Batra, 1999; Covey and Faure, 2005; Felix et al., 2011; Kopp-Scheinpflug et al., 2018) and appears to be the fundamental mechanism providing the extended delays required for pulse pattern recognition.

### Response dynamics of the non-spiking delay-line neuron LN5

PIR is fundamental to the operation of neural networks (Getting, 1989) it also has been implicated in functions akin to intrinsic short term memory (Goaillard et al., 2010). For the cricket delay-line neuron LN5 our data confirm the characteristic response of an inhibition followed by a rebound (Schöneich et al., 2015) and moreover show new response characteristics.

Upon stimulation with a sequence of sound pulses LN5 activity revealed the typical oscillation between inhibition and PIR. We additionally observed a gradual increase in its membrane potential over the time course of its response to normal chirps. This overall increase in response amplitude matched the tuning of the phonotactic behavior, and has not been described before. Due to these properties LN5 shows characteristics of pattern recognition, and it may not only provide a delayed excitation in form of the rebound depolarization but also an overall increased transmitter release to chirps with the species-specific pulse pattern. An increase of subsequent PIR amplitudes has been described in the stomatogastric ganglion of crabs for the lateral pyloric neurons. Over the time scale of seconds, the rebound in the pyloric neuron increases during repetitive stimulation with hyperpolarizing current pulses (Goaillard et al., 2010). The conductance(s) driving the amplitude change in PIR have not been worked out, but these experiments demonstrate an important plastic aspect of PIR generation.

In mammals PIR is initiated by strong glycinergic inputs and aided by the activation of hyperpolarization-activated cyclic nucleotide-modulated currents I_*h*_ and T-type calcium currents (Felix et al., 2011; Kopp-Scheinpflug et al., 2018), and in other systems, the conductance driving PIR has been identified as hyperpolarization activated inward current I_*h*_ (Pape, 1996). In the cricket auditory circuit conductances have not been identified, however, the time course of inhibition and depolarization may allow some insight into the currents driving the LN5 membrane potential. The inhibition at the start of the auditory response built up within 10 ms, matching the initial phasic response of LN2. At the start of the rebound, the conductance driving the depolarization appeared considerably stronger than the incoming inhibition from LN2. The LN5 rebound starts from the peak of the inhibition, although the inhibitory LN2 spike activity is still ongoing. Furthermore, the inhibition caused by a sound pulse was considerably smaller than the inhibition caused at a later stage of the rebound with a higher membrane potential. This may indicate that the current driving the rebound becomes considerably weaker as the rebound potential reaches its peak, and that the balance between the conductances driving the rebound depolarization and the inhibition shifts over the time course of the pulse intervals and chirp pattern. These two antagonistic conductances, may determine the dynamic of LN5 responses and its tuning, and may provide the basis for the overall increase of the LN5 membrane potential when stimulated with normal chirps.

In the I1- and I2-tests, for long time intervals the PIR increased over about 70 ms from the peak of the initial inhibition to reach its broad maximum. While our latencies and the timing of the inhibition elicited by the first sound pulse of a chirp are basically identical to the data of Schöneich et al. (2015), the development of the rebound over 63 ms for a 20 ms pulse appears to be 20 ms longer than the time previously given as (43 ms from the end of a pulse, Schöneich et al., 2015). The reason for this discrepancy is not clear. For long intervals our recordings reveal deflections in the rising phase, which slowed and delayed the development of the rebound. These were not coupled to the stimulus pattern and may indicate some additional inputs. This could have functional consequences for the coincidence detection process as for longer time intervals AN1 activity will coincide with a higher LN5 rebound amplitude and a stronger response of the coincidence detector should occur.

A computational modeling study demonstrates that LN5 response properties and its connection to LN3 rank very high in shaping the models response properties and can shape the tuning of the pattern recognition network (Clemens et al., 2020). We cannot yet explain the LN5 response differences in our I1/I2 experiments and tentatively point toward different LN5-like neurons. It is still not certain if the auditory brain neurons represent individuals or functional types. Surface electrode labeling of the ring-like auditory neuropil reveal three cluster with about 54 cell bodies, which could be linked to auditory processing (Kostarakos and Hedwig, 2017).

Behavioral studies (Poulet and Hedwig, 2005; Bent and Hedwig, 2021) reveal phonotaxis responses even toward non-attractive stimuli if these follow or are inserted into a sequence of calling song. This points toward a tolerant pattern recognition system, with an underlying plasticity of the circuitry and a modulatory effect over the time scale of the chirp pattern; and that the neural responses may depend on the order in which chirp patterns are presented (Doherty, 1985). The overall membrane potential response of LN5 demonstrates a tuning toward the species-specific pulse pattern, which occurs independent and before the coincidence-detection process, pointing toward a cell-specific intrinsic tuning of the LN5 response properties to the calling song pulse pattern that may have an impact on the response of the circuitry at a longer time scale. These response properties of LN5 could to be studied with sequences of excitatory current injections with the timing of the acoustic pulse pattern.

During phonotactic walking females steer to the very first pulse of a chirp, even before the pattern recognition process can be activated by that chirp (Hedwig and Poulet, 2004, 2005) and while the feature detector neuron LN4 is inhibited. Therefore, the pattern recognition process in the brain may not directly control the motor response of phonotactic steering, but rather seems to initiate and gate the phonotactic walking response (Poulet and Hedwig, 2005). Our data, demonstrating an increase on the LN5 membrane potential over the time course of a normal chirp which matched the phonotactic tuning would support such an organization of the behavior and could be coupled to a modulation of the output of the pattern recognition system.

### Pattern selectivity at the level of the coincidence-detector LN3 and feature detector LN4

Based on the network design the activity of coincidence detector neuron LN3 reflects the activity of AN1 and of the delay-line neuron LN5. For short pulse intervals LN3 generated an extended depolarization covering both sound pulses and did not resolve the pulse interval, like the spike activity in AN1. Recordings also revealed graded sub-threshold depolarizations of LN3 following its spiking activity, which likely represent input from the graded PIR depolarization of LN5 (Schöneich et al., 2015). LN3 also showed an overall depolarization of its membrane potential when exposed to normal chirp patterns, which may be linked to the increased depolarization of LN5 in response to normal chirps. As non-spiking interneurons (Pearson and Fourtner, 1975) will release transmitter in a graded way to drive postsynaptic neurons (Burrows and Siegler, 1976, 1978), the increased membrane potential of LN5 in response to a normal chirp could account for the gradual LN3 depolarization. Thus, the LN5 membrane potential may have an additional impact on the function and response property of the coincidence detector LN3, as indicated in the modeling study (Clemens et al., 2021). Furthermore, as expected from the coincidence-detector function, the spiking response to the second sound pulse of a normal chirp was always higher than the response to the first pulse (10). With longer time intervals the response amplitudes to a pulse gradually decreased again, reflecting the function of the delay-line circuit, and contributing to the tuning of the LN3 response (Schöneich et al., 2015).

The LN4 response to chirps is characterized by an initial hyperpolarization to the first sound pulse followed by sharp and short suprathreshold depolarization to each of the subsequent sound pulses (Kostarakos and Hedwig, 2012; Schöneich et al., 2015). When the interval between pulses was short and the response merged at the level of AN1 and LN3, so did the hyperpolarization of LN4 at the start of a chirp. With increasing pulse intervals, the excitation driven by the LN3 input is lost, and the response turns into an inhibition, corresponding to the LN4 activity reported by Kostarakos and Hedwig (2012). Thus, regarding pulse intervals, the processing in the pattern recognition circuity allows only a short time window, in which the feature detector generates an excitatory response. This is where the excitation provided from the coincidence detector LN3 overcomes the inhibition forwarded by LN2 (Kostarakos and Hedwig, 2012; Schöneich et al., 2015).

### Phonotaxis different levels: Linking pattern recognition and steering responses

Various hypothesis had been proposed to underlie cricket pattern recognition (Kostarakos and Hedwig, 2015). Our data are in line with the concept of a delay-line and coincidence detector network (Schöneich et al., 2015), demonstrating a tuning of the LN3 and LN4 neurons to the test patterns which match the tuning of the phonotactic behavior. Our recordings also confirm the sparse coding in the recognition pathway (Kostarakos and Hedwig, 2012) as the mean spiking response to a normal chirp drops by 85% from the response of AN1 to the response of LN4. Tuning to the pulse pattern occurs at low activity levels, with the observed tuning of the non-spiking neuron as a new feature of the circuit. The fundamental questions here are still left open: what is the output of the pattern recognition system and how does this neuronal activity scale to and drive the phonotactic behavior (Konishi, 1991)? By further exploring the link between high level *pattern recognition* and low level *auditory steering* which still remains poorly understood, we aim to answer these questions in the future.

## Data availability statement

The original contributions presented in this study are included in the article. Further inquiries can be directed to the corresponding author.

## Author contributions

XZ and BH: conceptualization, methodology, and writing—review and editing. XZ: formal analysis, investigation, and writing—original draft. BH: supervision and project administration. Both authors contributed to the article and approved the submitted version.
